# IVGTT-based simple assessment of glucose tolerance in the Zucker fatty rat: Validation against minimal models

**DOI:** 10.1371/journal.pone.0173200

**Published:** 2017-03-06

**Authors:** Micaela Morettini, Emanuela Faelli, Luisa Perasso, Sandro Fioretti, Laura Burattini, Piero Ruggeri, Francesco Di Nardo

**Affiliations:** 1 Department of Information Engineering, Università Politecnica delle Marche, Ancona, Italy; 2 Department of Experimental Medicine, University of Genoa, Genoa, Italy; University of Melbourne, AUSTRALIA

## Abstract

For the assessment of glucose tolerance from IVGTT data in Zucker rat, minimal model methodology is reliable but time- and money-consuming. This study aimed to validate for the first time in Zucker rat, simple surrogate indexes of insulin sensitivity and secretion against the glucose-minimal-model insulin sensitivity index (S_I_) and against first- (Φ_1_) and second-phase (Φ_2_) β-cell responsiveness indexes provided by C-peptide minimal model. Validation of the surrogate insulin sensitivity index (ISI) and of two sets of coupled insulin-based indexes for insulin secretion, differing from the cut-off point between phases (FPIR_3_-SPIR_3,_ t = 3 min and FPIR_5_-SPIR_5_, t = 5 min), was carried out in a population of ten Zucker fatty rats (ZFR) and ten Zucker lean rats (ZLR). Considering the whole rat population (ZLR+ZFR), ISI showed a significant strong correlation with S_I_ (Spearman’s correlation coefficient, r = 0.88; P<0.001). Both FPIR_3_ and FPIR_5_ showed a significant (P<0.001) strong correlation with Φ_1_ (r = 0.76 and r = 0.75, respectively). Both SPIR_3_ and SPIR_5_ showed a significant (P<0.001) strong correlation with Φ_2_ (r = 0.85 and r = 0.83, respectively). ISI is able to detect (P<0.001) the well-recognized reduction in insulin sensitivity in ZFRs, compared to ZLRs. The insulin-based indexes of insulin secretion are able to detect in ZFRs (P<0.001) the compensatory increase of first- and second-phase secretion, associated to the insulin-resistant state. The ability of the surrogate indexes in describing glucose tolerance in the ZFRs was confirmed by the Disposition Index analysis. The model-based validation performed in the present study supports the utilization of low-cost, insulin-based indexes for the assessment of glucose tolerance in Zucker rat, reliable animal model of human metabolic syndrome.

## Introduction

Insulin sensitivity and beta-cell function are tightly interconnected processes in the governed glucose tolerance [1,2]. To give a coordinated view of glucose disposal, a concomitant evaluation of quantitative indexes able to describe both phenomena is required. It is commonly acknowledged, indeed, that in the presence of a reduction of insulin sensitivity, glucose tolerance is maintained into the range of normality until beta-cells are unable to secrete an increased amount of insulin compensating for such reduction [3].

The investigation of the phenomena involved in the alteration of glucose tolerance is frequently performed in rodent models, among which the Zucker Fatty Rat (ZFR) is one of the most studied [4]. Interest in the ZFR relies on the fact that it is a well-recognized genetic model of human metabolic syndrome. This strain of rat is characterized by hyperinsulinaemia, glucose intolerance and insulin resistance [4,5].

Both in human and animal studies, the gold standard index for the quantification of insulin sensitivity is computed using the glucose clamp technique [6]. An equivalent estimation of insulin sensitivity [6] can be achieved by the interpretation of intravenous glucose tolerance test (IVGTT) data by minimal model of glucose kinetics (GKMM). With respect to glucose clamp, IVGTT requires simpler experimental procedures, thus allowing the wide application of this methodology both in humans [7–9] and in rats [10–12]. However, application is still limited to the study of quite small populations since running the GKMM is a non-trivial operation and requires a particular ability of the operator. In humans, simple surrogate indexes of insulin sensitivity/resistance in non-perturbed condition have been extensively validated against the gold standard and applied to large population studies [13]. However the same indexes have not provided satisfactory outcome in rodents, showing in rats, as in mice, modest correlation with the reference standard glucose clamp [14,15]. On the contrary, simple indexes of insulin sensitivity from IVGTT data have been introduced and validated both in man and in mice [16–18]. These indexes, based on glucose disappearance rate and on the area under the insulin curve, have never been adapted in rats.

Insulin secretion has been often quantitatively evaluated in rats and mice through indexes based on a dynamic (after a glucose perturbation) insulinaemia curve [19]. In particular, estimation of first-phase insulin response is commonly provided by the AIR_G_ index (Acute Insulin Response to Glucose) [20,21]. SPIR index (Second-Phase Insulin Response), based on the area under the curve of insulin, is used to estimate second-phase insulin response [22], although less frequently. The recent availability in the ZFR of C-peptide data during an IVGTT allowed a reliable estimation of first- and second-phase insulin secretion through the definition of a minimal model of C-peptide kinetics (CPMM) [12]. From the physiological standpoint, C-peptide is preferable to insulin. Indeed, C-peptide is secreted with insulin in equimolar concentrations from the beta-cells but, differently from insulin, is not affected by degradation operated by the liver. Despite a greater accuracy in estimating insulin secretion, the application of a methodology based on C-peptide measurements is discouraged by the high costs of the C-peptide measurements kits. Furthermore, the approach with the CPMM suffers the previously mentioned disadvantages of the model-based procedures. Although insulin-based AIR_G_ and SPIR indexes are commonly applied both in man and rat, to our knowledge no study has been designed so far to adapt and validate these indexes in rats.

On this basis, the aim of the study is to validate simple surrogate insulin sensitivity and insulin-based beta-cell function indexes against minimal-model-based indexes, in order to provide a coordinated assessment of glucose tolerance in the Zucker rat. Validation was performed by direct comparison with insulin sensitivity provided by the GKMM and the first and second phase beta-cell responsiveness indexes provided by the CPMM.

## Materials and methods

### Animals

This study included 20 male Zucker rats (Charles River Laboratories), divided into 2 groups: a group of 7-to-9week-old homozygous fatty rats (ZFR, fa/fa, n = 10) and a group of age-matched heterozygous lean rats (ZLR, fa/+, n = 10). All rats were housed in controlled conditions of temperature (21±1°C), humidity (60±10%) and lighting (08.00–20.00 h) and received a standard rat chow containing 0.3% sodium, with tap water *ad libitum*. The experiments were performed at 08.00 h, after a 12 h overnight fast. The animals were anesthetized with sodium pentobarbital (50 mg·kg^-1^ i.p., plus maintenance doses if necessary; Sigma Chemical, St. Louis, Missouri, USA). In our laboratory experience [23] this anaesthetic has shown to be adequate, since it does not alter insulin secretion, and artefactual dose-dependent effects are not seen. The adequacy of the anaesthesia was assessed by monitoring the changes in heart rate (HR) and mean arterial pressure (MAP) and by the state of the pupils. Changes in MAP and HR are the most valuable indicators of adequacy of the depth of anaesthesia [24]. The onset of instability in heart rate and arterial pressure were assumed as indicators of inadequacy of the depth of anaesthesia.

The experiments were performed in accordance with Italian national guidelines on animal experimentation (Decreto Legislativo 27/1/1992, no. 116, Attuazione della Direttiva no. 86/609/CEE in materia di protezione degli animali utilizzati a fini sperimentali o ad altri fini scientifici). The study was approved by the Ethical committee of IRCCS S. Martino-IST (Comitato per la sperimentazione etica sugli animali), Genoa, Italy (Permit n. 253). Rectal temperature was controlled and maintained at 37.5±0.5°C by a heating pad. The right femoral artery and vein were cannulated. The arterial cannula, connected to a pressure transducer (Spectramed Statham P23XL, Viggo-Spectramed, Oxnard, California, USA) provided a recording of AP through a Grass preamplifier, model 7P14A (Grass Instruments, Quincy, Massachusetts, USA). HR was monitored using a Grass tachograph (model 7P4), triggered by lead II of the electrocardiogram (ECG). The venous cannula was used for drug injection. AP, ECG and HR were digitally recorded by an A/D converter (CED Power1401, Cambridge Electronic Design, Cambridge, UK), stored on a PC and analysed by laboratory software (Spike2, CED). At the end of the experiments the animals were sacrificed by an overdose of sodium pentobarbital.

### Intravenous Glucose Tolerance Test (IVGTT)

Two basal blood samples (200 μL) were taken from the arterial catheter at –5 and –2 min before glucose injection. A glucose bolus of 400 mg·kg^-1^ was then injected over 1 min into the femoral vein (conventional time-zero). The volume of the glucose bolus initially given was 400 μL, that matched the volume withdrawn in the two basal blood samples. Ten additional blood samples (200 μL) were collected at 1, 2, 3, 5, 8, 15, 25, 40, 70 after the injection, for the measurement of glucose, insulin and C-peptide concentration. Plasma volume was replaced by normal saline infusion matching the blood volume withdrawn for the sampling.

### Assays

Blood was promptly centrifuged and glucose immediately measured with the glucose oxidase method using an automated glucose analyser. The remaining plasma was stored at –80°C for later insulin determination. Insulin and C-peptide were measured with commercially available rat insulin and rat C-peptide ELISA kits (Mercodia, Uppsala, Sweden). The sensitivity of the insulin assay is 11.7 pmol·L^-1^, with an inter-and intra- assay precision of 3.3 and 1.8 respectively. The sensitivity of the C-peptide assay is 27.5 pmol·L^-1^ with an inter-and intra- assay precision of 2.9 and 4.4 respectively.

### Model-based indexes

#### Insulin sensitivity

Insulin sensitivity index S_I_ (min^-1^/(pmol·L^-1^)) was estimated by applying the GKMM [25] to fit glucose data. A non-linear least squares estimation technique [26] implemented in SAAM II software [27] was used. Insulin data were assumed as error-free model input and were linearly interpolated for the simulation. Errors in glucose measurements were assumed to be uncorrelated, Gaussian, zero mean. The procedure, has been described in detail in authors’ previous work [28].

#### β-cell responsiveness

First- and second-phase β-cell responsiveness indexes, Φ_1_ and Φ_2_, were estimated by fitting C-peptide data using the CPMM. Such model was originally introduced for humans by Cobelli and Pacini [29] and adapted for Zucker rats in a recent work by the authors of the present paper [12].

The indexes Φ_1_ and Φ_2_ are defined as follows:

$$\Phi_{1}=\frac{{\text{CP}}_{0}}{\Delta \text{G}}$$ (1)

$$\Phi_{2}=\frac{\partial^{2}({\text{SR}}_{2}(\text{t}))}{\partial \text{G}\partial \text{t}}=\frac{\partial^{2}(\gamma \cdot (\text{G}(\text{t})-\text{h})\cdot \text{t})}{\partial \text{G}\partial \text{t}}=\gamma .$$ (2)

Φ_1_ ((pmol/L C-peptide)/(mmol/L glucose)) measures the incremental amount of C-peptide, CP_0_, (per unit volume of compartment 1) released during the first phase of β-cell response normalized to the maximum increment, ΔG, of plasma glucose concentration after the injection (defined as the difference between the peak, G_max_, and the steady-state, G_ss_, value of plasma glucose concentration). The index Φ_2_ (min^-2^(pmol/L C-peptide)/(mmol/L glucose)) describes stimulatory effect of glucose concentration on provision into the β-cells and release of new insulin. This index is expressed as second order partial derivatives of second phase release, SR_2_(t), with respect to glucose and time. G(t)–h (mmol/L) is the deviation of plasma glucose concentration G(t) from a threshold level, h and *t* is the time interval which follows the glucose injection.

Secretion parameters γ, and CP_0_ were estimated in each rat by fitting to measured C-peptide data. A non-linear least squares estimation technique [26] implemented in SAAM II software [27] was used. Glucose data were assumed as error-free model input and were linearly interpolated for the simulation. Errors in C-peptide measurements were assumed to be uncorrelated, Gaussian, zero mean. The responsiveness indexes Φ_1_ and Φ_2_, were subsequently computed for individual cases. Further details are reported in authors’ previous work [12].

### Surrogate indexes

#### Insulin sensitivity

As shown by Bergman [30], the insulin sensitivity index depends on the glucose disappearance rate and the suprabasal insulin concentration following the glucose stimulation. On this basis, a surrogate simple assessment of insulin sensitivity [17,18] could be performed in the Zucker rat by the following insulin sensitivity index (ISI) index:

$$\text{ISI}=\frac{{\text{K}}_{\text{G}}}{{\text{AUC}}_{\text{D}}}$$ (3)

where K_G_ is the intravenous glucose tolerance index and AUC_D_ is the mean above-basal area under insulin curve in the interval 0–70 min. K_G_ was computed as -100·b, where b is the slope of the logarithm of glucose concentration vs. time in the interval detected as the main elimination phase (between minute 3 and minute 25). AUC_D_ is computed as the area under the suprabasal insulin curve (I(t)-I_70_) according to the trapezoidal rule, divided by the length of the interval (70 min). Measurement units of ISI are min^-1^/(pmol·L^-1^).

#### β-cell responsiveness

First phase insulin release index (FPIR) and second phase insulin release index (SPIR) were considered for the description of first and second phase secretion, respectively. Two different formulations for each one of the two empiric indexes were tested, in relation to the cut-off point assumed for the discrimination of first-phase end and second-phase beginning. In the first formulation, we considered that first-phase secretion ends 5 minutes after glucose infusion. Thus, first-phase insulin secretion was calculated as the mean increment above basal of insulinaemia values measured at 1, 2, 3 and 5 min after intravenous glucose bolus [20], normalized to fasting glycaemia (FPIR_5_). SPIR index accounting for the second-phase insulin responsiveness was computed as the incremental area of insulin from 5 to 70 min after intravenous glucose bolus, using the trapezoidal rule, normalized to fasting glycaemia (SPIR_5_).

In the second formulation, as suggested by other authors, the end of the first phase was set 3 minutes after glucose infusion [21]. The corresponding formulation of first-phase insulin response (FPIR_3_) was computed as the mean increment above basal of insulinaemia values measured at 1, 2, and 3 min after intravenous glucose bolus, normalized to fasting glycaemia. Thus, second-phase insulin responsiveness (SPIR_3_) was computed as the incremental area of insulin from 3 to 70 min after intravenous glucose bolus, normalized to fasting glycaemia. The normalization to fasting glycaemia was performed to obtain indexes of responsiveness more easily comparable with CPMM indexes (Φ_1_ and Φ_2_).

#### Disposition index

In the present study, the following expression [1] of the Disposition Index (DI) was used for the characterization of glucose tolerance in Zucker rats using surrogate indexes:

DI=ISI⋅FPIR (4)

### Statistical analysis

The Lilliefors test was used to evaluate the hypothesis that each data vector or parameter vector had a normal distribution with unspecified mean and variance. Comparisons between two groups of normally distributed samples were performed with two-tailed, unpaired Student’s *t* test; Wilcoxon rank sum test was used to compare samples which were not normally distributed. To quantify the linear regression analysis, Pearson’s product-moment correlation coefficient and Spearman's rank correlation coefficient were used for normally and non-normally distributed populations, respectively. Significance level was set at 5%.

To evaluate the degree of agreement between each pair of surrogate and model-based index, Bland-Altman plots and Lin’s concordance correlation coefficient were computed. Since surrogate and model-based indexes do not provide the same numerical value, each surrogate index has been corrected by means of the regression curve against the corresponding model-based index, which corresponds to the best estimate of the functional relation between the two measurements.

## Results

### Measurements

Mean or median values for age, body weight, fasting glycaemia, insulinaemia and C-peptide for the two groups of Zucker rat are reported in Table 1. With respect to age-matched ZLR group, the ZFR group shows significantly higher body weight as well as fasting glycaemia, insulinaemia and C-peptide.

**Table 1:** Characteristics of our groups of Zucker rats.

Variable	ZLR	ZFR	Statistics
	(n = 10)	(n = 10)	
Age (wk)	8 [1]	8.4 [1]	NS**
BW (g)	230 ± 9	289 ±7	*P* < 0.001*
Fasting glycaemia (mmol·L^-1^)	4.33 ± 0.28	6.0 ± 0.22	*P* < 0.001*
Fasting insulinaemia (pmol·L^-1^)	78 [122]	686 ± 115	*P* < 0.001**
Fasting C-peptide (pmol·L^-1^)	235 [382]	2846 ± 440	*P* < 0.001**

Values are means ±SE or median [IQR]. ZLR, Zucker Lean Rat; ZFR, Zucker Fatty Rat; BW, body weight; NS, not significant;

* Unpaired Student’s t-test;

** Wilcoxon rank sum test.

**Fig 1:**
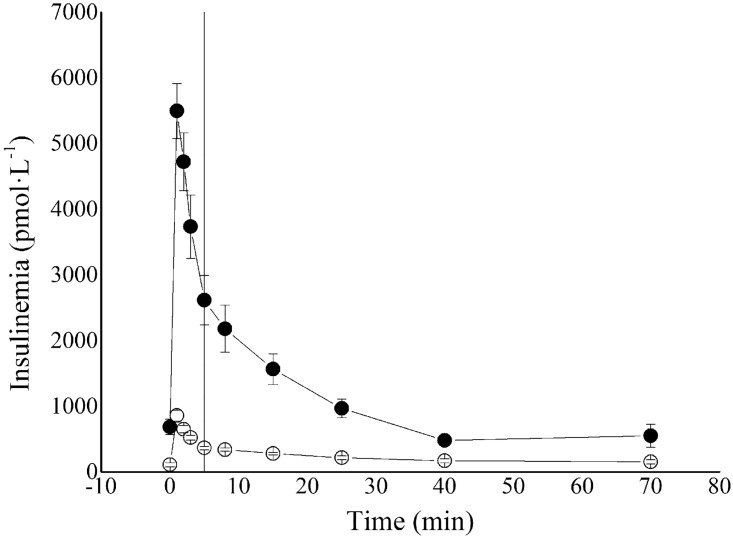
Average insulinaemia profiles (± SE) for ZLR (open circles) and ZFR (closed circles) group. Vertical line represented the cut-off point between first- and second-phase secretion (5^th^ min).

**Fig 2:**
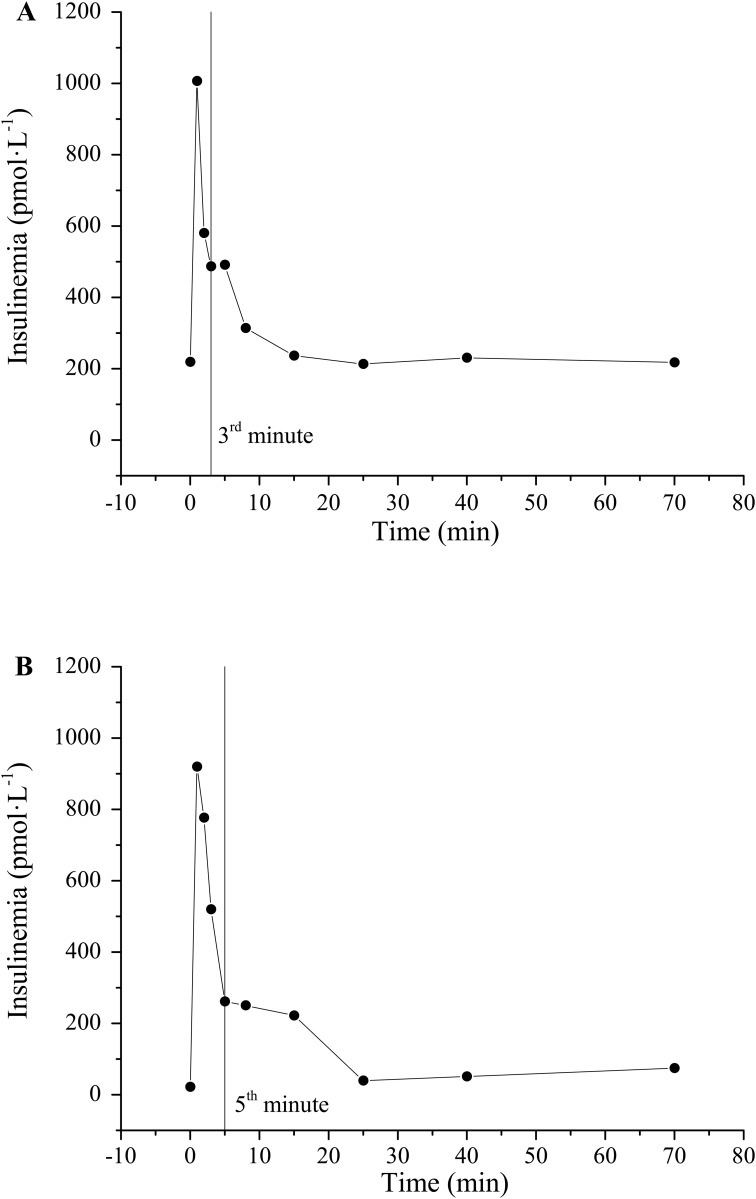
Insulinaemia profiles for two ZLRs from control population. Vertical line represented the cut-off point between first- and second-phase secretion (3^rd^ min in panel A and 5^th^ min in panel B).

The average insulinaemia curve for ZLRs and ZFRs is reported in Fig 1. Representative examples of cut-off point between first- and second-phase secretion at 3^rd^ and 5^th^ minute are reported in Fig 2A and 2B respectively.

### Model-based indexes

Mean or median estimates of model-based insulin sensitivity, and first and second- phase responsiveness indexes for the two groups of rats are reported in Table 2. On average, S_I_ showed significantly lower values (83%) in the ZFR group with respect to ZLR group. Indexes of both first and second-phase responsiveness showed significantly higher median values in the ZFR group with respect to ZLR group. In particular, the relative increase observed in the median values was 255% in Φ_1_ and 392% in Φ_2_.

**Table 2:** Model-based indexes in ZLR and ZFR groups.

	INSULIN SENSITIVITY	FIRST-PHASE β-CELL RESPONSIVENESS	SECOND-PHASE β-CELL RESPONSIVENESS
	S_I_	Φ_1_	Φ_2_
ZLR	1.7 [1] (20%)	186 ± 13 (22%)	1.60 [1.47] (37%)
ZFR	0.3 [0.1] (28%)	661 ± 98 (19%)	7.87 ± 0.52 (37%)
Statistics	*P* < 0.001**	*P* < 0.001*	*P* < 0.001**

ZLR, Zucker Lean Rat; ZFR, Zucker Fatty Rat; S_I_ (10^−4^·min^-1^/(pmol·L^-1^)); Φ_1_ ((pmol/L C-peptide)/(mmol/L glucose)); Φ_2_ (10^−1^ min^-2^·(pmol/L C-peptide)/(mmol/L glucose)). Values are reported as means ± SE or as median [IQR]. The percent coefficient of variation of the estimates (CV%) was given in parentheses.

* Unpaired Student’s t-test;

** Wilcoxon rank sum test.

### Surrogate indexes

**Fig 3:**
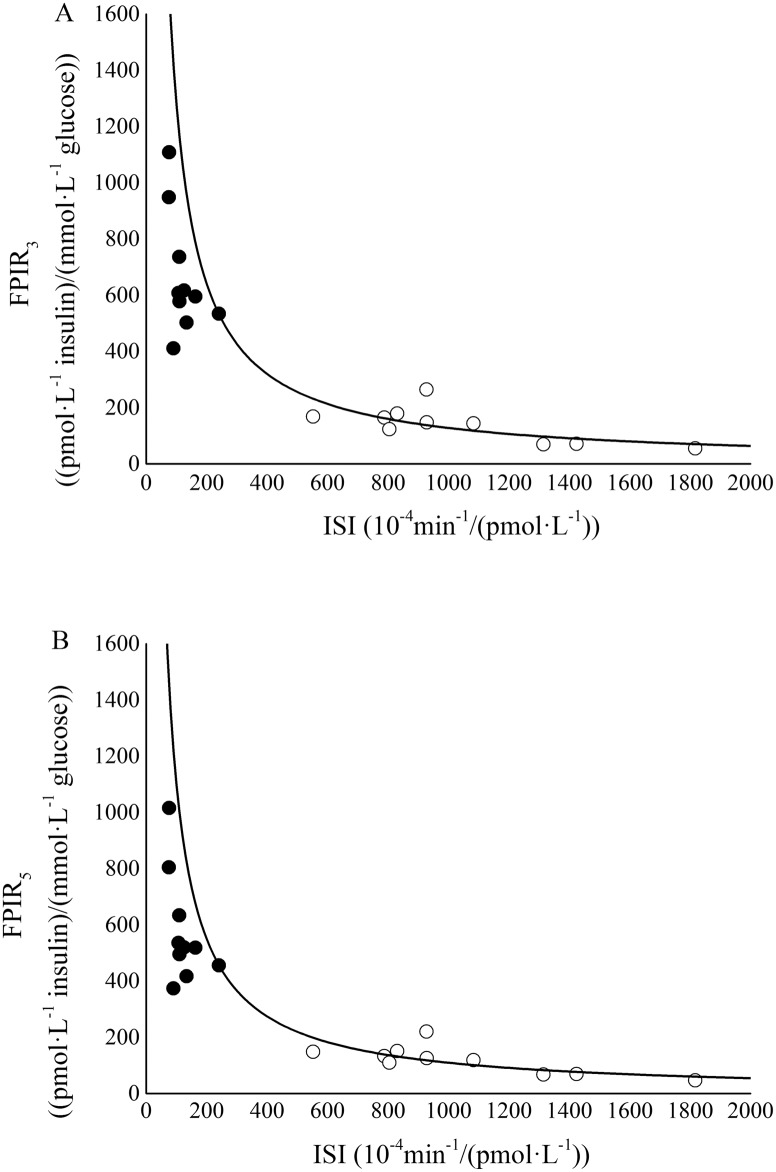
FPIR_3_ vs ISI (panel A) and FPIR_5_ vs ISI (panel B) for ZLRs (open circles) and ZFRs (closed circles). Hyperbolic line was obtained by the best fitting procedure in ZLRs.

Mean or median estimates of surrogate insulin sensitivity, and first and second- phase responsiveness indexes for the two groups of rats are reported in Table 3. On average, ISI showed significantly lower values (88%) in the ZFR group with respect to ZLR group. Indexes of both first and second-phase responsiveness showed significantly higher median values in the ZFR group with respect to ZLR group. In particular, the relative increase observed in the median values was 333% in FPIR_3_, 336% in FPIR_5_, 213% in SPIR_3_, and 193% in SPIR_5_. Disposition index was also computed in each single rat (Fig 3). Significantly lower mean values for DI_3_ and DI_5_ were observed in the ZFR group with respect to ZLR group (Table 3). In particular, the relative reduction observed in the mean values was 41% in DI_3_ and 40% in DI_5_.

**Table 3:** Surrogate indexes in ZLR and ZFR groups.

	INSULIN SENSITIVITY	FIRST-PHASE β-CELL RESPONSIVENESS		SECOND-PHASE β-CELL RESPONSIVENESS		DISPOSITION INDEX	
	ISI	FPIR_3_	FPIR_5_	SPIR_3_	SPIR_5_	DI_3_	DI_5_
ZLR	1047 ± 118	139 ± 20	119 ± 16	3.1 ± 0.3	2.9 ± 0.3	13.0 ± 1.5	11.2 ± 1.1
ZFR	123 ± 16	602 [161]	519 [144]	9.7 ± 1.0	8.5 ± 0.9	7.7 ± 0.7	6.7 ± 0.6
Statistics	*P* < 0.001*	*P* < 0.001**	*P* < 0.001**	*P* < 0.001*	*P* < 0.001*	*P* < 0.05*	*P* < 0.05*

ZLR, Zucker Lean Rat; ZFR, Zucker Fatty Rat; ISI (10^−4^·min^-1^/(pmol·L^-1^)); FPIR ((pmol/L insulin)/(mmol/L glucose)); SPIR (10^5^ min·(pmol/L insulin)/(mmol/L glucose)). DI (min^-1^/(mmol/L glucose)). Values are reported as means ± SE or as median [IQR].

* Unpaired Student’s t-test;

** Wilcoxon rank sum test

### Correlation and agreement between model-based and surrogate indexes

**Fig 4:**
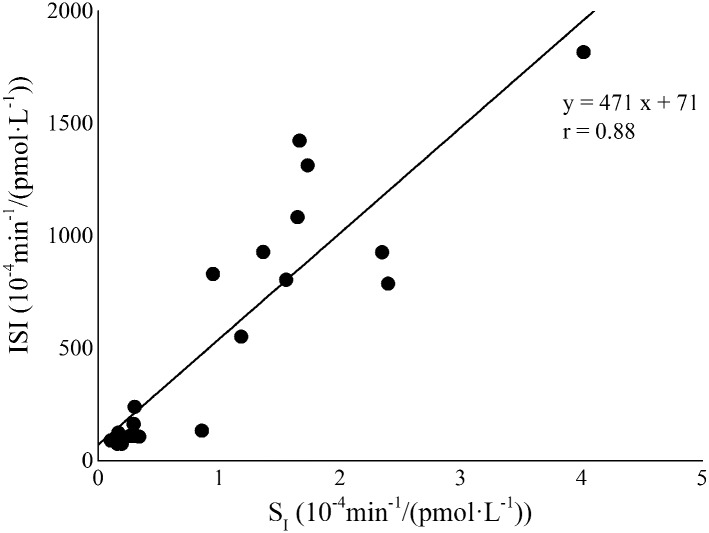
Correlation between ISI and S_I_ in the whole rat population (ZLR+ZFR).

**Fig 5:**
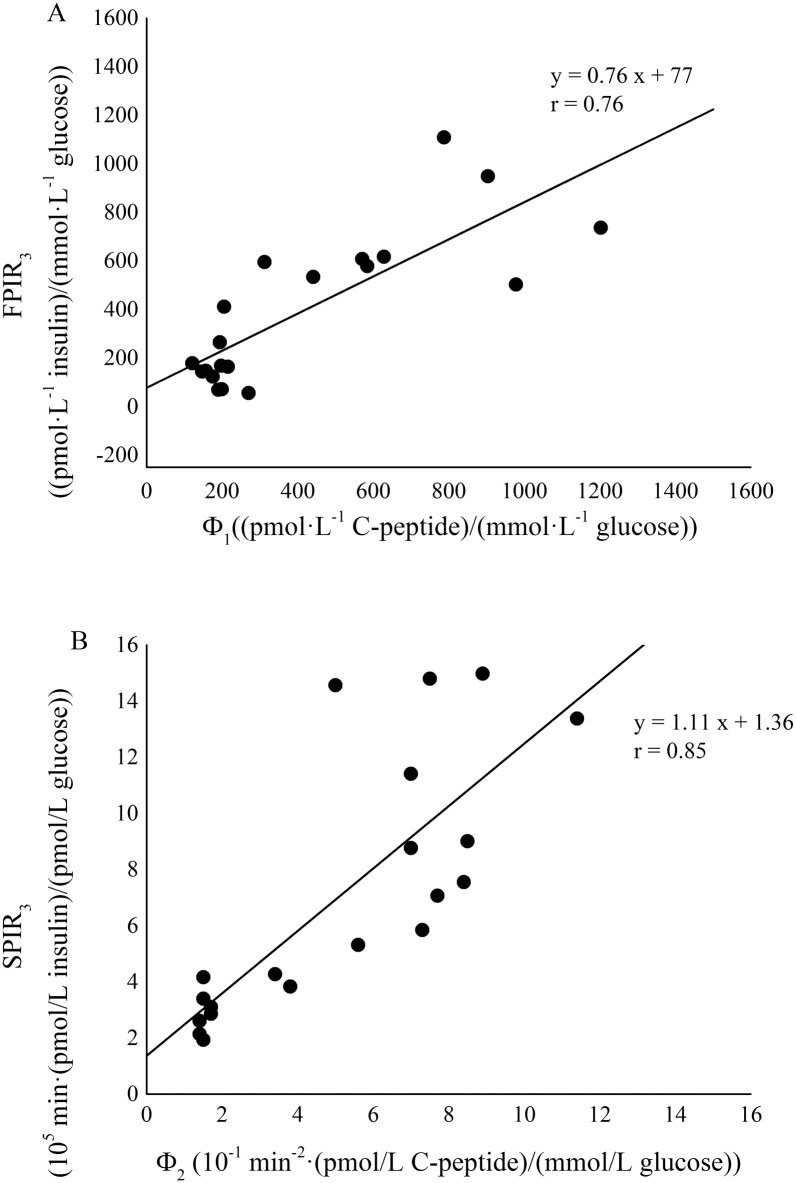
Correlation between FPIR_3_ and Φ_1_ (panel A) and between SPIR_3_ and Φ_2_ (panel B) in the whole rat population (ZLR+ZFR).

**Fig 6:**
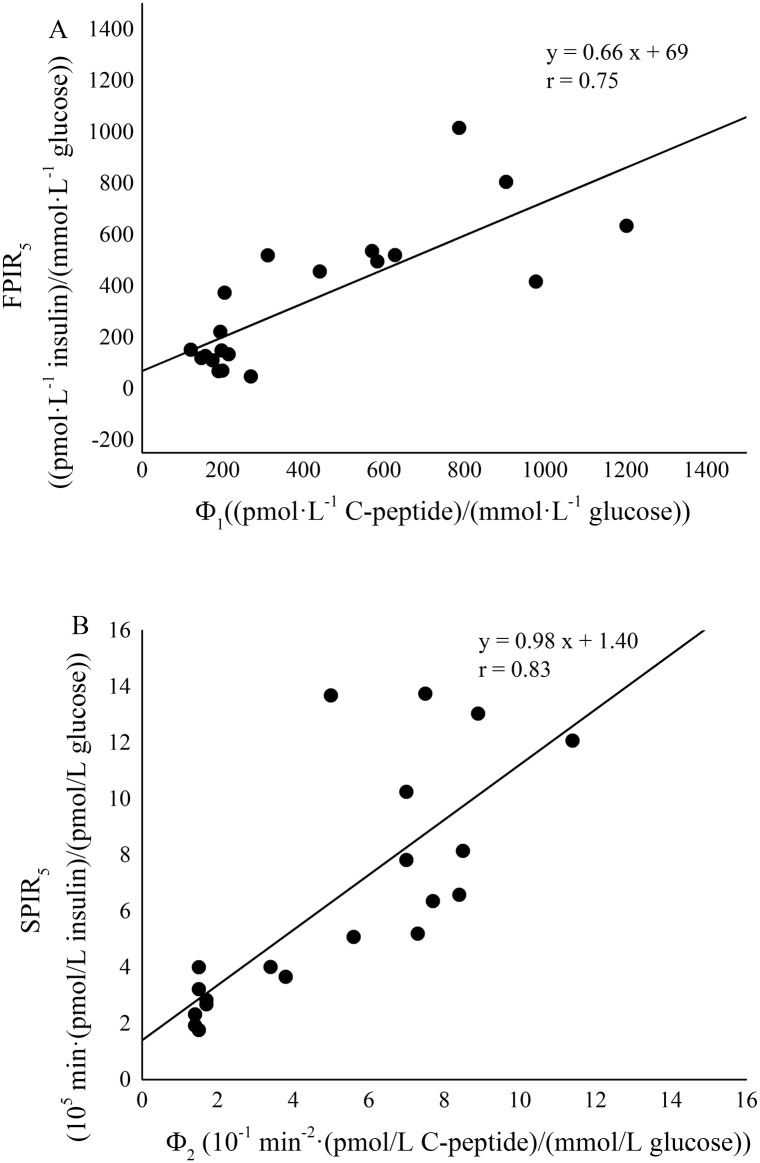
Correlation between FPIR_5_ and Φ_1_ (panel A) and between SPIR_5_ and Φ_2_ (panel B) in the whole rat population (ZLR+ZFR).

A significant positive linear correlation between ISI and S_I_ values was detected in the whole rat population (Fig 4, Spearman’s correlation coefficient, r = 0.88 and P < 0.001). The two formulations of FPIR index, FPIR_3_ and FPIR_5_ significantly correlated each other (Spearman’s correlation coefficient, r = 0.998 and P < 0.001). Likewise, the correlation between SPIR_3_ and SPIR_5_ was very strong and significant (Pearson’s correlation coefficient, r = 0.997 and P < 0.001). A significant positive linear correlation was detected in the whole rat population between FPIR_3_ and Φ_1_ (Fig 5, panel A, Spearman’s correlation coefficient, r = 0.76 and P < 0.001) and between SPIR_3_ and Φ_2_ (Fig 5, panel B, Spearman’s correlation coefficient, r = 0.85 and P < 0.001). Similarly, FPIR_5_ and the corresponding second-phase responsiveness index SPIR_5_ showed a significant linear correlation with Φ_1_ (Fig 6, panel A, Spearman’s correlation coefficient, r = 0.75 and P < 0.001) and Φ_2_ (Fig 6, panel B, Pearson’s correlation coefficient, r = 0.83 and P < 0.001), respectively.

**Fig 7:**
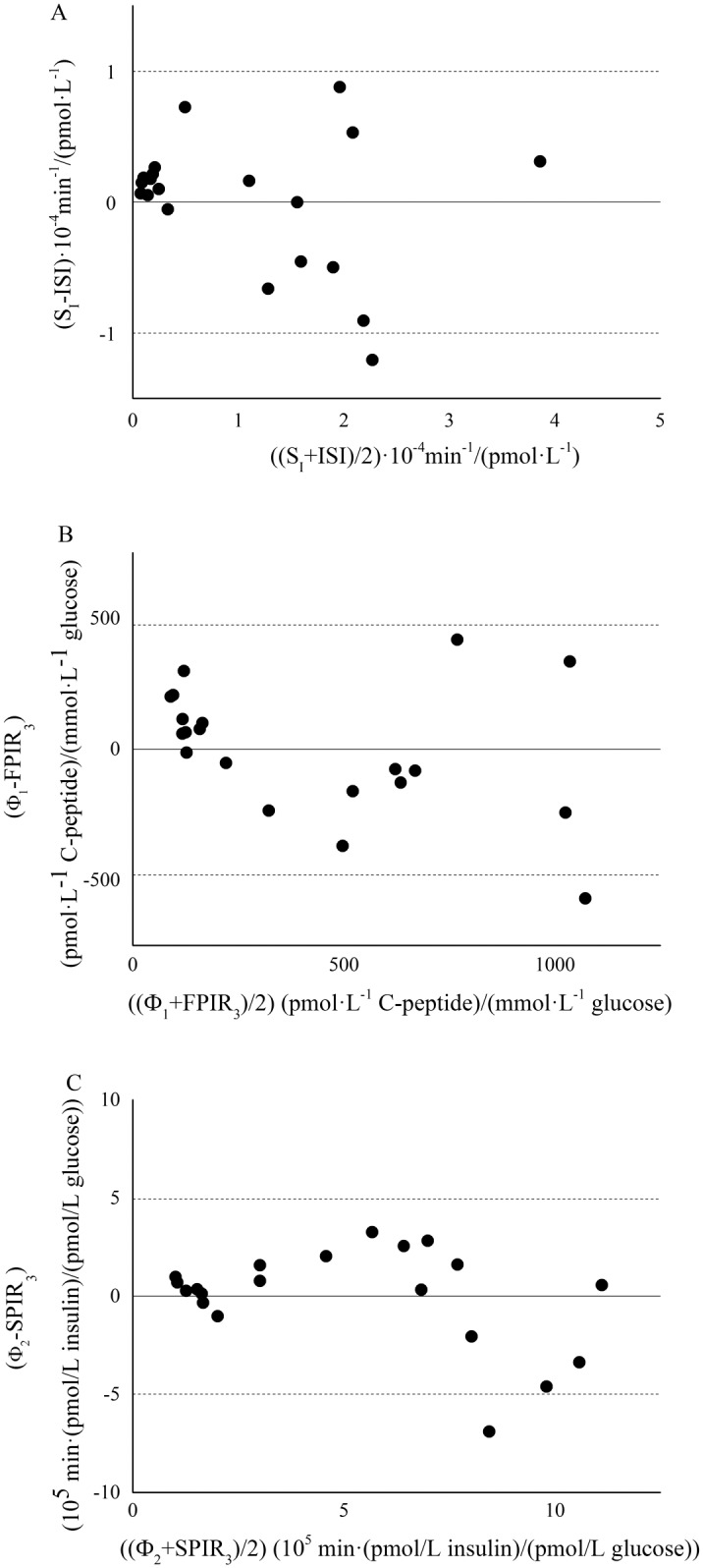
Bland-Altman plots for corrected ISI and S_I_ (panel A), corrected FPIR_3_ and Φ_1_ (panel B), corrected SPIR_3_ and Φ_2_ (panel C). Dashed lines represents limit of agreement (mean ± 1.96 SD).

Agreement between each pair of surrogate and model-based indexes, was tested by means of Lin’s concordance correlation coefficient and Bland-Altman plots. Lin’s concordance correlation coefficient is 0.89 for ISI vs. SI, 0.78 for FPIR vs. Φ1, and 0.76 for FPIR vs. Φ2, respectively. Bland-Altman plots are reported in Fig 7 (panels A, B and C). Reported results for insulin responsiveness surrogate indexes, considered 3rd minute as the cut-off point; similar results were obtained with cut-off at 5th minute (data not shown).

## Discussion

The present study aimed at validating, against minimal-model methodologies, IVGTT-based indexes for a simple assessment of glucose tolerance in the Zucker Fatty Rat. In order to exhaustively determine the degree of glucose tolerance, insulin sensitivity should be assessed in association with beta-cell responsiveness. In the present study, an index (ISI) for the simple assessment of insulin sensitivity from IVGTT data in rats was evaluated by using an approach similar to that developed in mice [17], assuming that insulin sensitivity could be provided by the ratio between K_G_ and insulin AUC during the whole duration of the IVGTT test. ISI was validated in the whole population by direct comparison with GKMM S_I_, showing a significant and strong correlation with the model-based index (Spearman’s correlation coefficient, r = 0.88, P < 0.001). The elevated value of correlation suggests that ISI is able to provide an accurate and reliable assessment of insulin sensitivity in the ZFR.

For the evaluation of first-phase secretion the well-known AIR_G_ index (Acute Insulin Response to Glucose) was considered as a basis. AIR_G_ has been quantified in a variety of ways in literature. The most common formulation considered the mean value of suprabasal insulin samples between 1 and 5 min [19,20,31]. A further useful definition considered the mean value of suprabasal insulin samples between 1 and 3 min [21]. The analysis of the insulinaemia curve in every single rat, as shown in representative examples in Fig 2, demonstrated that first-phase end varied within the interval 3–5 minutes. For these reasons, in the present paper both 3^rd^ and 5^th^ minute were tested as cut-off points. Moreover, they were normalized to basal glycaemia to obtain FPIR index which have the same units as CPMM-based index Φ_1_. FPIR_3_ and FPIR_5_ were validated in the whole population by direct comparison with Φ_1_. Significant and strong correlation with the model-based index were found for both FPIR_3_ (r = 0.76, P < 0.001) and FPIR_5_ (r = 0.75, P < 0.001). Similar correlation with Φ_1_ were expected for FPIR_3_ and FPIR_5_ since these two indexes showed a significant strong correlation each other (Pearson’s correlation coefficient, r = 0.998, P < 0.001). Some authors suggested to normalize AIR_G_ to the incremental glucose peak [32] but, in our population of Zucker rats, this resulted in a worse correlation with Φ_1_ (results not shown). Recently, a formulation of AIR_G_ between 0 and 4 minutes has been proposed as an index of insulin secretion in un-anaesthetized mice [33]. Since the present IVGTT protocol lacks of the sample at minute 4, at present was not possible to test performance of this index in Zucker Rat. However, the non-significant difference observed between FPIR_3_ and FPIR_5_ in characterizing insulin secretion, suggested that selecting minute 4 as the cut-off point would not lead to different results.

As for the first-phase, SPIR_3_ and SPIR_5_ were validated in the whole population by direct comparison with model-based index (Φ_2_). Significant and strong correlation with Φ_2_ were found for both SPIR_3_ (r = 0.85, P < 0.001) and SPIR_5_ (r = 0.83, P < 0.001). Similar correlation with Φ_2_ were expected for SPIR_3_ and SPIR_5_ since these two indexes showed a significant strong correlation each other (Pearson’s correlation coefficient, r = 0.997, P < 0.001). Differently from first-phase indexes, the units of SPIR_3_ and SPIR_5_ are not the same as Φ_2_ but normalization allows to obtain an index of beta-cell function and not only of insulin release, thus making the comparison with Φ_2_ more appropriate.

The elevated values of correlation between insulin-based and CPMM-based indexes suggest that FPIR and SPIR indexes are able to provide an accurate and reliable assessment of insulin secretion in the ZFR. The strong correlation detected between FPIR_3_ and FPIR_5_ (r = 0.998, P < 0.001) and between SPIR3 and SPIR_5_ (r = 0.997, P < 0.001) indicates that FPIR_3_-SPIR_3_ and FPIR_5_-SPIR_5_ provide similar information in relation to beta-cell responsiveness to glucose. In an effort to find a standardization of the cut-off sample separating first from second-phase, FPIR_3_ and SPIR_3_ seem to provide slightly better correlations with Φ_1_ and Φ_2_, respectively. Agreement between each pair of surrogate and model-based indexes was confirmed by the elevated values of Lin’s concordance correlation coefficient (not lower than 0.76) and by Bland-Altman plots, as reported in Fig 7.

In the present study, the recognized existence of an insulin-resistant state in ZFR was confirmed by a significant reduction of ISI with respect to ZLR (123 ± 16 vs. 1047 ± 118 10^−4^·min^-1^/(pmol·L^-1^), P < 0.001). A concomitant significant increase in beta-cells responsiveness was detected in ZFR by means of first-phase (FPIR_3_ and FPIR_5_) and second-phase (SPIR_3_ and SPIR_5_) indexes (Table 3). This matches with the well-known presence of hyperinsulinemia in the ZFR strain [5] and with the enhancement of both first- and second-phase secretion assessed in ZFR by CPMM interpretation of C-peptide data [12]. The analysis of glucose tolerance in insulin resistant rats was achieved by laying ZFR sensitivity-secretion data on the hyperbolic function found in the control group, ZLR (Fig 3). The ZFR sensitivity-secretion data (closed circles in Fig 3) laid on the early portion of the hyperbolic curve, indicating a substantial defect in insulin action. However, the distribution of ZFR sensitivity-secretion data around the hyperbolic curve suggested that hypersecretion was able to partially compensate the insulin resistant state. As long as this compensation is adequate, i.e. the disposition index does not change, the glucose tolerance is not impaired. The present data (Table 3) showed a significant decrease in mean DI values in ZFRs. This suggested that the increase of insulin secretion was starting to become inadequate in relation to the degrees of insulin resistance and glucose intolerance was arising. Nevertheless, glycaemia values did not overcome the diabetes threshold in ZFR (Table 1) and thus type 2 diabetes did not develop yet. These results further support the accuracy and reliability of the surrogate indexes in describing glucose tolerance in Zucker Fatty Rat.

## Conclusion

The model-based validation performed in the present study supports the utilization of the surrogate indexes for the assessment of glucose tolerance in the animal model of human metabolic syndrome, the Zucker fatty rat. Results showed, indeed, that the considered indexes are able to provide an easy, low-cost but accurate assessment of insulin sensitivity and beta-cell function in the ZFR. Although not intended to replace the minimal-model methodologies, these indexes offer important advantages in estimating insulin sensitivity and biphasic insulin secretion in population studies. Since the main advantages over model-based and C-peptide-based indexes rely on simplicity in computation and lower costs of measurements, these indexes could be even more suitable in studies over a very large number of animals.

## Abbreviations

Φ_1_: First-phase β-cell responsiveness derived from CPMM
Φ_2_: Second-phase β-cell responsiveness derived from CPMM
CPMM: C-Peptide Kinetics Minimal Model
DI: Disposition Index
FPIR: First-phase Insulin Release, i.e. Surrogate First-phase β-cell responsiveness index
GKMM: Glucose Kinetics Minimal Model
ISI: Surrogate Insulin sensitivity index
IVGTT: Intravenous Glucose Tolerance Test
S_I_: Insulin sensitivity index derived from GKMM
SPIR: Second-phase Insulin Release, i.e. Surrogate Second-phase β-cell responsiveness index
ZFR: Zucker Fatty Rat
ZLR: Zucker Lean Rat
